# HO-1/CO Maintains Intestinal Barrier Integrity through NF-*κ*B/MLCK Pathway in Intestinal HO-1^−/−^ Mice

**DOI:** 10.1155/2021/6620873

**Published:** 2021-05-19

**Authors:** Zhenling Zhang, Lijing Zhang, Qiuping Zhang, Bojia Liu, Fang Li, Yi Xin, Zhijun Duan

**Affiliations:** ^1^Department of Gastroenterology, The First Affiliated Hospital of Dalian Medical University, Dalian 116011, China; ^2^Department of Pathology, The First Affiliated Hospital of Dalian Medical University, Dalian 116011, China; ^3^Department of Immunology, Dalian Medical University, Dalian 116044, China; ^4^Department of Biochemistry and Molecular Biology, Dalian Medical University, Dalian 116044, China

## Abstract

**Background:**

Intestinal barrier injury is an important contributor to many diseases. We previously found that heme oxygenase-1 (HO-1) and carbon monoxide (CO) protect the intestinal barrier. This study is aimed at elucidating the molecular mechanisms of HO-1/CO in barrier loss.

**Materials and Methods:**

We induced gut leakiness by injecting carbon tetrachloride (CCl_4_) to wildtype or intestinal HO-1-deficient mice. In addition, we administrated tumor necrosis factor-*α* (TNF-*α*) to cells with gain- or loss-of-HO-1 function. The effects of HO-1/CO maintaining intestinal barrier integrity were investigated *in vivo* and *in vitro*.

**Results:**

Cobalt protoporphyrin and CO-releasing molecule-2 alleviated colonic mucosal injury and TNF-*α* levels; upregulated tight junction (TJ) expression; and inhibited epithelial I*κ*B-*α* degradation and phosphorylation, NF-*κ*B p65 phosphorylation, long MLCK expression, and MLC-2 phosphorylation after administration of CCl_4_. Zinc protoporphyrin completely reversed these effects. These findings were further confirmed *in vitro*, using Caco-2 cells with gain- or loss-of-HO-1-function after TNF-*α*. Pretreated with JSH-23 (NF-*κ*B inhibitor) or ML-7 (long MLCK inhibitor), HO-1 overexpression prevented TNF-*α*-induced TJ disruption, while HO-1 shRNA promoted TJ damage even in the presence of JSH-23 or ML-7, thus suggesting that HO-1 dependently protected intestinal barrier via the NF-*κ*B p65/MLCK/p-MLC-2 pathway. Intestinal HO-1-deficient mice further demonstrated the effects of HO-1 in maintaining intestinal barrier integrity and its relative mechanisms. Alleviated hepatic fibrogenesis and serum ALT levels finally confirmed the clinical significance of HO-1/CO repairing barrier loss in liver injury.

**Conclusion:**

HO-1/CO maintains intestinal barrier integrity through the NF-*κ*B/MLCK pathway. Therefore, the intestinal HO-1/CO-NF-*κ*B/MLCK system is a potential therapeutic target for diseases with a leaky gut.

## 1. Introduction

In the intestine, the epithelial barrier that regulates the interaction between the luminal material (e.g., gut microbiome) and the interstitium (e.g., mucosal immune cells) is crucial for maintaining homeostasis. The intestinal epithelial barrier function is critical for selective gut permeability and limits the entry of bacteria and pathological bacterial components like lipopolysaccharide (LPS) from the intestinal lumen to the body [1]. If the epithelium is intact, the intestinal barrier function is largely defined by tight epithelial junction (TJ) proteins, such as the transmembrane protein occludin [2] and the peripheral membrane protein zonula occludens 1 (ZO-1) [3]. Mild or severe disruption of the intestinal epithelial barrier can enhance or directly trigger inflammatory bowel disease (IBD) [4], colorectal carcinoma [5], or liver diseases [6]. Therefore, repairing intestinal barrier loss is essential in preventing or delaying the progression of such diseases.

Heme oxygenase-1 (HO-1), a stress-inducible enzyme, catalyzes the initial and rate-limiting step in the oxidative degradation of heme, yielding equimolar amounts of biliverdin IX*α* (BV), carbon monoxide (CO), and free iron [7]. CO-releasing molecule 2 (CORM-2) can spontaneously transfer CO and exert typical CO-mediated pharmacological effects [8]. Recent *in vivo* and *in vitro* studies have demonstrated that the HO-1-CO axis prevents intestinal barrier dysfunction [9, 10]. HO-1 and CO can prevent intestinal inflammation in mice by promoting bacterial clearance [11]. In our previous study, we identified that HO-1 dependently preserves the intestinal mucosal barrier integrity by abrogating TJ dysregulation and epithelial cell damage [12]. We also found that HO-1 elevation ameliorates intestinal barrier function in bile duct ligation- (BDL-) induced cholestatic liver injury by inhibiting NF-*κ*B p65 [13]. However, whether NF-*κ*B p65 directly mediates the intestinal TJ protein dysregulation still remains unclear.

The myosin light-chain kinase (MLCK) participates in intestinal barrier dysfunction [14]. MLCK has two splice variants derived from the same gene using different promoters. Short or smooth muscle MLCK is not expressed in the intestinal epithelium, whereas long MLCK is highly expressed in intestinal epithelial cells and regulates TJ permeability by inducing phosphorylation of myosin light-chain 2 (MLC-2) [14–16], which, in turn, leads to remodeling of the TJ structure. Tumor necrosis factor *α* (TNF-*α*) has been shown to promote TJ dysregulation and induce epithelial barrier loss by elevating the expression and activity of long MLCK [17, 18]. This elevation is in part mediated by NF-*κ*B [19, 20], and a few *κ*B sites have been identified in the upstream promoter region that specifically drives long MLCK activation [21, 22].

Hence, based on our previous findings, in this study, we induced gut dysfunction (leakage) by injecting carbon tetrachloride (CCl_4_) to wildtype (WT) or intestinal HO-1-deficient mice or by administrating TNF-*α* to HO-1 overexpression or knockdown cells. The effects of HO-1/CO maintaining intestinal barrier integrity were examined *in vivo* and *in vitro*. These data may provide new ideas for the targeted regulation of intestinal epithelial barrier integrity.

## 2. Materials and Methods

### 2.1. Animal Experiments

C57BL/6 male WT mice (6-8 weeks of age and weighing 20-25 g) were obtained from the Laboratory Animal Center of Dalian Medical University (Liaoning, China). The intestinal HO-1 conditional knockout (HO-1^−/−^) mice were constructed using C57BL/6 mice by the Beijing Viewsolid Biotechnology Co. Ltd. (Beijing, China). All the animals were housed in an environment with a temperature of 22 ± 1°C, a relative humidity of 50 ± 1%, and a light/dark cycle of 12/12 hr and fed with food and water *ad libitum*. All animal studies (including the mice euthanasia procedure) were done in compliance with the regulations and guidelines of Dalian Medical University institutional animal care and conducted according to the AAALAC and the IACUC guidelines (approval No. AEE18006).

The mouse gut leakiness model was induced by CCl_4_ (Shanghai Aladdin Biochemical Technology Co., Ltd., Shanghai, China) [23]. Briefly, the mice were administered 2 mL/kg CCl_4_ by intraperitoneal injection (CCl_4_ : olive oil = 1 : 3) twice a week for 12 weeks. The control group was given olive oil. In the last 2 weeks, according to their groupings, all surviving mice were administered cobalt protoporphyrin (CoPP, 5 mg/kg, Sigma-Aldrich, USA), zinc protoporphyrin (ZnPP, 5 mg/kg, Sigma-Aldrich), CORM-2 (8 mg/kg, Sigma-Aldrich), or inactivated-CORM-2 (iCORM-2, 8 mg/kg) by intraperitoneal injection twice a week for 2 weeks [12]. For the control and CCl_4_ groups, the mice received an intraperitoneal injection of saline. iCORM-2 was generated as previously described by incubation overnight (18 h) at 37°C and bubbling with air (N_2_) to remove the residual CO [24]. The WT C57BL/6 mice were randomized into six groups: control (*n* = 6), CCl_4_ (*n* = 10), CCl_4_+CoPP (*n* = 12), CCl_4_+ZnPP (*n* = 12), CCl_4_+CORM-2 (*n* = 13), and CCl_4_+iCORM-2 (*n* = 10).

VillinCre Hmox1^floxp/floxp^ mice with conditional knockout HO-1 in the intestinal epithelial cells were obtained by crossing VillinCre transgenic mice with Hmox1^floxp/floxp^ mice containing Loxp sites flanking exon 2 of the *hmox1* gene (Supplementary Figure S3) [12]. WT and Hmox1^floxp/floxp^ mice were bred and used as controls for experiments involving VillinCre Hmox1^floxp/floxp^ mice. WT, Hmox1^floxp/floxp^, and VillinCre Hmox1^floxp/floxp^ mice were administered CCl_4_ to establish the gut leakage mice model. The mice were randomized to six groups: WT-Control (*n* = 5), Hmox1^floxp/floxp^-Control (*n* = 5), VillinCre Hmox1^floxp/floxp^-Control (*n* = 6), WT-CCl_4_ (*n* = 10), Hmox1^floxp/floxp^-CCl_4_ (*n* = 5), and VillinCre Hmox1^floxp/floxp^-CCl_4_ (*n* = 10).

At the end of the experiment, mice were sacrificed by cervical dislocation, and blood, colon, and liver samples were collected. The serum samples were obtained by centrifugation of the blood at 2,500 × g for 10 min. The serum levels of alanine aminotransferase (ALT) were determined using a commercial kit (Nanjing Jiancheng Biotechnology Institute, Nanjing, China), according to the manufacturer's instructions. The isolated colon tissue concentrations of TNF-*α* were measured using the ELISA kits (Wuhan USCN Business Co., Ltd., Wuhan, China) following the manufacturer's protocols. The colon and liver tissues in each group were fixed with 10% paraformaldehyde for histopathological staining, and the remnants of colon tissues were stored at -80°C for later use.

### 2.2. Cell Culture

The human colonic adenocarcinoma cell line Caco-2 was cultured in Dulbecco's modified Eagle's medium (DMEM, Gibco, Invitrogen Inc., Carlsbad, CA, USA) supplemented with 10% fetal calf serum (FCS, Gibco), 100 U/mL penicillin, and 100 mg/mL streptomycin at 37°C in a 5% CO_2_ atmosphere. The cells were separately transfected with the human FUGW-HO-1 and pLKO.1-sh-HO-1 plasmids (Hanheng Biotechnology Corp., Shanghai, China) to overexpress and knockdown HO-1, respectively. Lipofiter™ (Hanbio Biotechnology, China) was used for transfection, according to the manufacturer's instructions. In separate experiments, the cells were pretreated with JSH-23 (10 *μ*M, Selleckchem, Houston, TX, USA), a specific inhibitor of NF-*κ*B for 3 h [13], or ML-7 (10 *μ*M, Selleckchem), an inhibitor of long MLCK for 3 h [25], followed by 24 h of TNF-*α* (100 ng/mL, PeproTech, Rocky Hill, NJ, USA) [21] treatment to simulate intestinal epithelial barrier damage.

### 2.3. Western Blot

Isolated colonic epithelia and cell monolayers were sonicated in RIPA lysis buffer (Beyotime Biotechnology, Shanghai, China) with protease and phosphatase inhibitors (Biotool, Houston, USA) and centrifuged at 14,000 × g for 15 min. The supernatants were then transferred to a new enzyme-free tube. Protein concentrations were determined by using the bicinchoninic acid protein assay kits (Beyotime Biotechnology). Samples were mixed with 4x loading buffer and run on an 8% or 10% sodium dodecyl sulfate-polyacrylamide gel. The proteins were transferred to a polyvinylidene fluoride membrane at 250 mA for 2 h. The membranes were blocked for nonspecific binding in 5% milk in TBS-Tween 20 (TBST) for 1 h at room temperature and then incubated overnight at 4°C with anti-ZO-1 (1 : 500; Proteintech Cat# 21773-1-AP; RRID:AB_10733242), anti-occludin (1 : 1000; Abcam Cat# ab167161; RRID:AB_2756463), anti-I*κ*B-*α* (1 : 1000; Abcam Cat# ab32518; RRID:AB_733068), anti-phospho-I*κ*B-*α* (1 : 1000; Abcam Cat# ab133462; RRID:AB_2801653), anti-NF-*κ*B p65 (1 : 500; Cell Signaling Technology Cat# 6956; RRID:AB_10828935), anti-phospho-NF-*κ*B p65 (1 : 500; Cell Signaling Technology Cat# 3033; RRID:AB_331284), anti-long MLCK (1 : 1000; Abcam Cat# ab76092; RRID:AB_1524000), anti-MLC-2 (1 : 500; Cell Signaling Technology Cat# 3672; RRID:AB_10692513), anti-phospho-MLC-2 (1 : 500; Cell Signaling Technology Cat# 3675; RRID:AB_2250969), anti-*β*-actin (1 : 2000; Proteintech Cat# 60008-1-Ig; RRID:AB_2289225), or anti-GAPDH (1 : 2000; Proteintech Cat# 60004-1-Ig, RRID:AB_2107436). Membranes were then washed in TBST for 30 min, exposed to the secondary antibody linked to horseradish peroxidase for 1 h, and washed for 30 min in TBST before being developed using ECL detection reagents (Millipore Corp., Billerica, MA, USA).

### 2.4. Hematoxylin and Eosin, Mayer-Sirius Red Staining, and Immunohistochemistry

The paraffin-embedded colon and liver samples were used to prepare 5 *μ*m thick sections with a microtome. The sections were stained with hematoxylin and eosin (H&E) using standard methods. For Mayer-Sirius red collagen staining, the liver sections were deparaffinized and stained with Sirius red buffer for 1 h at room temperature. After washing, the sections on the slides were stained with Mayer solution and mounted. Immunohistochemistry (IHC) staining was performed according to standard methods. All of these slides were examined and read by an experienced pathologist who was blinded to the study design. The Image J software was used to analyze the images of Sirius red and IHC staining.

Colonic epithelial injury from H&E staining was scored according to the inflammatory manifestations and lesion depths of the colon [26]. Inflammatory manifestations (1-4 points) are as follows: 1 = mild inflammation and scattered mononuclear cells can be seen focally; 2 = moderate inflammation with scattered mononuclear cells in many places; 3 = severe inflammation, accompanied by increased vascular density and significantly thickened intestinal wall; and 4 = extreme inflammation, accompanied by a full layer of leukocyte infiltration of the intestinal wall and disappearance of goblet cells. Lesion depths (0-3 points) are as follows: 0 = none; 1 = submucosa; 2 = muscle layer; and 3 = serious film layer.

### 2.5. Data Analysis

All presented data were representative of three or more independent experiments, each with similar results. The continuous data are shown as mean ± standard deviation. Comparisons between the two groups were performed using Student's *t*-test. Comparisons among multiple groups were made using ANOVA of Tukey's post hoc test. *P* values ≤ 0.05 were considered statistically significant.

## 3. Results

### 3.1. Damage to the Intestinal Mucosal Barrier and Activation of the NF-*κ*B p65/MLCK Pathway Is Abolished after CoPP and CORM-2 Treatment following CCl_4_ Injection

**Figure 1:**
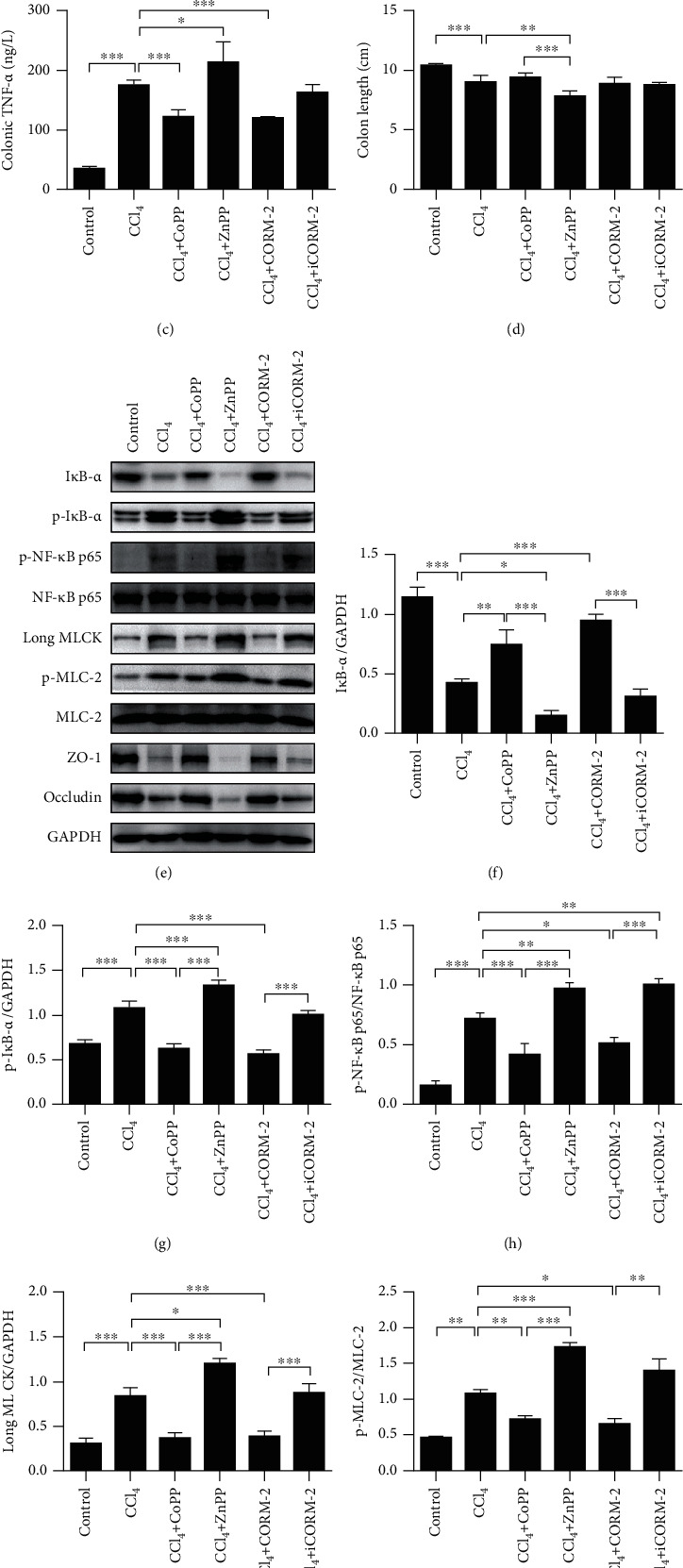
CoPP and CORM-2 mice show less barrier loss and NF-*κ*B p65/MLCK pathway activation following CCl_4_ injection. C57BL/6 wildtype mice received CCl_4_ injection for 12 weeks. All surviving mice were then administrated with CoPP, ZnPP, CORM-2, or iCORM-2 for the last 2 weeks. (a) Representative colon sections after hematoxylin and eosin (H&E) staining (200x, scale bar = 10 *μ*m). (b) Colon pathology scores. (c) Colonic TNF-*α* levels (*n* = 5). (d) Colon length (cm) (*n* = 5). (e) Representative protein bands and quantification analyses of a Western blot for (f) I*κ*B-*α*, (g) p-I*κ*B-*α*, (h) p-NF-*κ*B p65/NF-*κ*B p65, (i) long MLCK, (j) p-MLC-2/MLC-2, (k) ZO-1, and (l) occludin in the colonic epithelia. All presented data were representative of three or more independent experiments, each with similar results. ^∗^*P* < 0.05, ^∗∗^*P* < 0.01, and ^∗∗∗^*P* < 0.001.

To define the role of HO-1/CO in repairing intestinal barrier loss, C57BL/6 WT mice were subjected to 12 weeks of CCl_4_ injection. All surviving mice (about 75% survival rate) were then administrated with CoPP (an HO-1 inducer), ZnPP (an HO-1 inhibitor), CORM-2, or iCORM-2 for the last 2 weeks. Western blot and qRT-PCR were used to confirm that HO-1 protein and mRNA were upregulated in colonic epithelia after applying CoPP, but not with ZnPP (Supplementary Figure S1a and S1b). Next, we investigated whether HO-1 and CO are involved in regulating intestinal epithelial barrier integrity following CCl_4_ injection. The increased pathological score and TNF-*α* levels of the colon (Figures 1(a)–1(c)), the reduced length of the colon (Figure 1(d)), and the disrupted proteins of TJs such as ZO-1 (Figures 1(e) and 1(k)) and occludin (Figures 1(e) and 1(l)) were observed in the CCl_4_-treated group (all *P* < 0.001). CoPP treatment attenuated the CCl_4_-induced colon pathological changes (*P* < 0.05) and TNF-*α* levels (*P* < 0.001) but did not affect the colon length (*P* > 0.05) (Figures 1(a)–1(d)). Importantly, CoPP and CORM-2 administration significantly increased the expression of colonic epithelial ZO-1 (both *P* < 0.001) and occludin (both *P* < 0.01) proteins as compared to the CCl_4_-treated group (Figures 1(e), 1(k), and 1(l)). ZnPP treatment significantly promoted colonic TNF-*α* levels (Figure 1(c), *P* < 0.05) and decreased the length of the colon and the expression of colonic epithelial TJ proteins (ZO-1 and occludin) after CCl_4_ administration (Figures 1(d), 1(e), 1(k), and 1(l), all *P* < 0.01). However, ZnPP did not affect colonic mucosal damage after CCl_4_ challenge in mice (Figures 1(a) and 1(b), *P* > 0.05). In addition, there was no significant difference in the pathological scores, TNF-*α* levels, length of the colon, and expression of colonic epithelial ZO-1 and occludin between CCl_4_ and iCORM-2 mice (Figures 1(a)–1(e), 1(k), 1(l), all *P* > 0.05). Our findings suggested that the upregulation of HO-1 and CO may repair intestinal epithelial barrier injury after CCl_4_ injection.

On the other hand, CCl_4_ induced a significant degradation of the inhibitor of nuclear factor-*κ*B *α* (I*κ*B-*α*), which was consistent with a marked upregulation of the phosphorylation levels of I*κ*B-*α* and NF-*κ*B p65 in colonic epithelial tissues of mice as compared to the control group (Figures 1(e)–1(h), all *P* < 0.001). As expected, CCl_4_ dramatically induced long (epithelial) MLCK expression (Figures 1(e) and 1(i), *P* < 0.001) and MLC-2 phosphorylation (but not MLC-2 level) (Figures 1(e) and 1(j), *P* < 0.01), compared with the control group. Interestingly, CoPP and CORM-2 treatment significantly inhibited I*κ*B-*α* degradation and phosphorylation, reduced NF-*κ*B p65 activity and phosphorylation, and suppressed long MLCK activation and MLC-2 phosphorylation compared to the CCl_4_-treated group (Figures 1(e)–1(j)). ZnPP treatment completely reversed these effects as a response to CCl_4_ administration (Figures 1(e)–1(j)). In addition, iCORM-2 significantly upregulated NF-*κ*B p65 phosphorylation, but it had no effects on the degradation and phosphorylation of I*κ*B-*α* and on the expression of long MLCK and phospho-MLC-2 after CCl_4_ administration (Figures 1(e)–1(j)). Taken together, these findings indicated that the NF-*κ*B p65/MLCK-p-MLC-2 pathway might be the crucial downstream molecular mechanism of the HO-1-CO axis on protecting against intestinal barrier loss after CCl_4_ injection.

### 3.2. Elevation of HO-1 in Intestinal Epithelial Monolayer Cells Protects against Barrier Loss after TNF-*α* Stimulation

**Figure 2:**
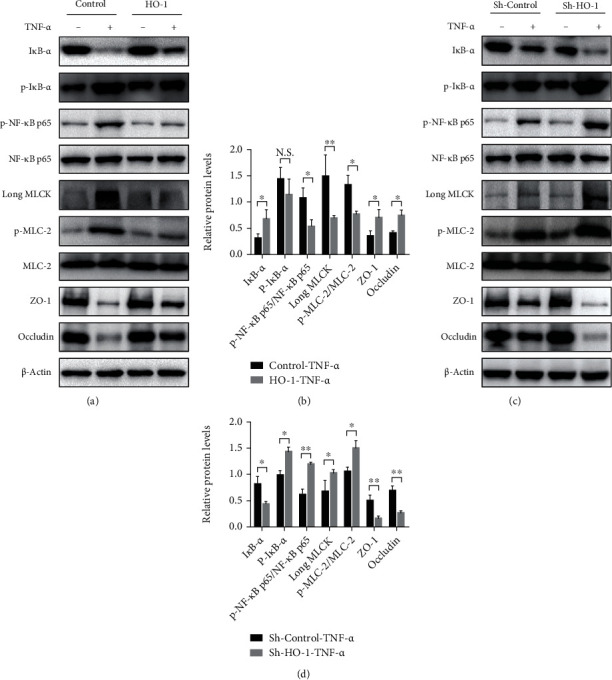
HO-1 overexpression protects against epithelial barrier loss after TNF-*α* stimulation. (a, b) Representative protein bands and quantification analyses of a Western blot for I*κ*B-*α*, p-I*κ*B-*α*, p-NF-*κ*B p65/NF-*κ*B p65, long MLCK, p-MLC-2/MLC-2, ZO-1, and occludin in Caco-2 cells transfected with the FUGW-HO-1 plasmid after TNF-*α* stimulation. (c, d) Representative protein bands and quantification analyses of a Western blot for I*κ*B-*α*, p-I*κ*B-*α*, p-NF-*κ*B p65/NF-*κ*B p65, long MLCK, p-MLC-2/MLC-2, ZO-1, and occludin in Caco-2 cells transfected with the pLKO.1-sh-HO-1 plasmid after TNF-*α* stimulation. All data presented are representative of three or more independent experiments, each with similar results. ^∗^*P* < 0.05 and ^∗∗^*P* < 0.01. N.S.: no significance.

In order to confirm the in vivo studies of HO-1 repairing intestinal barrier injury, gain- or loss-of-HO-1-function experiment was conducted using Caco-2 cells transfected with FUGW-HO-1 or pLKO.1-sh-HO-1 plasmid *in vitro*. The cells transfected with empty plasmids served as the control groups (Supplementary Figure 2). Similar to the *in vivo* data, the expression of epithelial ZO-1, occludin, and I*κ*B-*α* was significantly increased (all *P* < 0.05), while the expression of phospho-NF-*κ*B p65 (*P* < 0.05), long MLCK (*P* < 0.01), and phospho-MLC-2 (*P* < 0.05) were remarkably decreased in Caco-2 cells transfected with FUGW-HO-1 plasmid after TNF-*α* treatment, compared to the scrambled control group (Figures 2(a) and 2(b)). However, relative to the control group, HO-1 shRNA significantly decreased the expression of epithelial ZO-1 (*P* < 0.01), occludin (*P* < 0.01), and I*κ*B-*α* (*P* < 0.05) proteins and increased the expression of phospho-I*κ*B-*α* (*P* < 0.05), phospho-NF-*κ*B p65 (*P* < 0.01), long MLCK (*P* < 0.05), and phospho-MLC-2 (*P* < 0.05) as a response to TNF-*α* treatment (Figures 2(c) and 2(d)). These data suggested that HO-1 dependently repairs intestinal barrier dysfunction, followed by reduced NF-*κ*B p65/MLCK-p-MLC-2 pathway activation.

### 3.3. HO-1 Overexpression and NF-*κ*B p65 Signaling in Intestinal Epithelial Cells Are Required for Regulating Barrier Loss following TNF-*α* Stimulation

**Figure 3:**
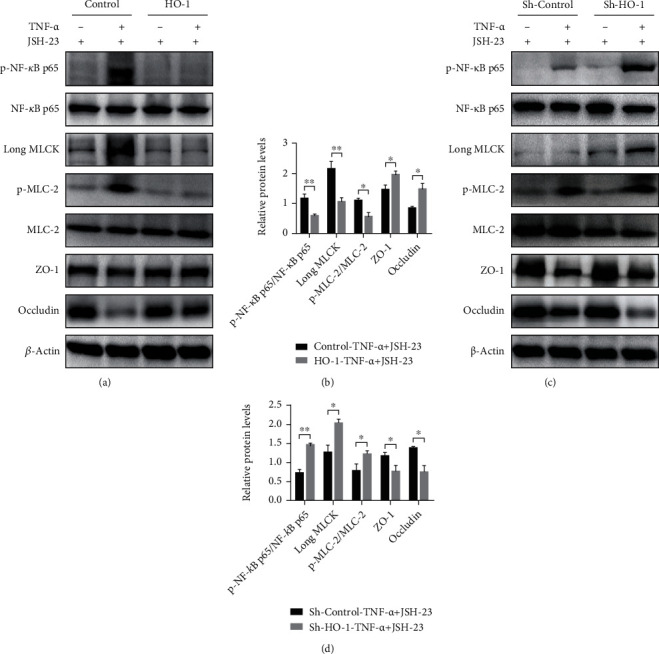
The barrier function is dependent on HO-1 and NF-*κ*B p65 signals from intestinal epithelial cells. (a, b) Representative protein bands and quantification of the Western blot for p-NF-*κ*B p65/NF-*κ*B p65, long MLCK, p-MLC-2/MLC-2, ZO-1, and occludin in Caco-2 cells transfected with FUGW-HO-1 plasmid after pretreatment of JSH-23 with or without TNF-*α* stimulation. (c, d) Representative protein bands of a Western blot for p-NF-*κ*B p65/NF-*κ*B p65, long MLCK, p-MLC-2/MLC-2, ZO-1, and occludin in Caco-2 cells transfected with pLKO.1-sh-HO-1 plasmid after pretreatment of JSH-23 with or without TNF-*α* stimulation. All data presented were representative of three or more independent experiments, each with similar results. ^∗^*P* < 0.05 and ^∗∗^*P* < 0.01.

To investigate whether NF-*κ*B p65 mediated the downstream MLCK-p-MLC-2 signaling in intestinal epithelial cells and its relationship with HO-1 and barrier loss, we used JSH-23, a specific NF-*κ*B inhibitor, to pretreat Caco-2 cells in which HO-1 function was either increased or decreased. The results showed that in the presence of JSH-23, HO-1 overexpression significantly reduced NF-*κ*B p65 phosphorylation, long MLCK expression, and MLC-2 phosphorylation and markedly increased ZO-1 and occludin expression in Caco-2 cells transfected with the FUGW-HO-1 plasmid after TNF-*α* stimulation, compared to the scrambled control group (Figures 3(a) and 3(b)). In contrast, even in the presence of JSH-23, Caco-2 cells transfected with the pLKO.1-sh-HO-1 plasmid showed higher phosphorylation of NF-*κ*B p65, expression of long MLCK, and phosphorylation of MLC-2 and lower expression of ZO-1 and occludin after TNF-*α* stimulation, compared to the scrambled control group (Figures 3(c) and 3(d)). These findings indicated that NF-*κ*B p65 might mediate the activation of epithelial long MLCK, the phosphorylation of MLC-2 in enterocytes, and the disruption of TJs, and it may also contribute to reduced barrier function. Importantly, HO-1 dependently blocked the activation of NF-*κ*B p65 and downstream MLCK-p-MLC-2 signaling, resulting in intestinal epithelial barrier function restoration.

### 3.4. HO-1 Elevation and Long MLCK Inhibition in Intestinal Epithelial Cells Are Protected against Barrier Loss after TNF-*α* Stimulation

**Figure 4:**
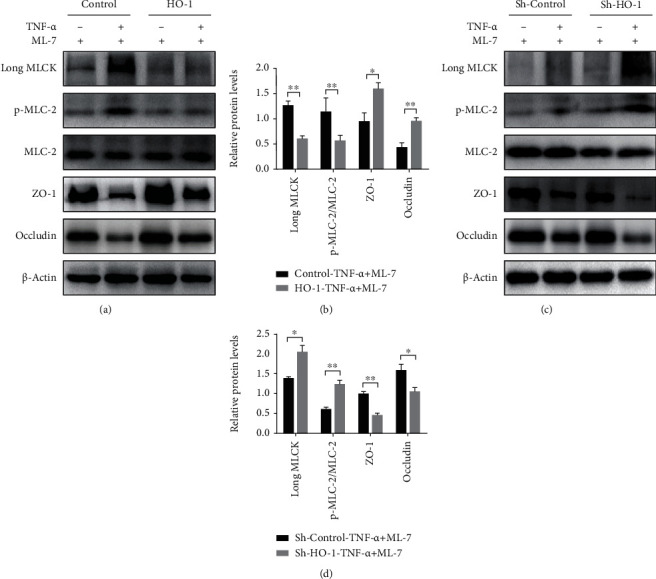
HO-1 and MLCK in intestinal epithelial cells mediate barrier loss. (a, b) Representative protein bands and quantification analyses of a Western blot for long MLCK, p-MLC-2/MLC-2, ZO-1, and occludin in Caco-2 cells transfected with FUGW-HO-1 plasmid after pretreatment of ML-7 with or without TNF-*α* stimulation. (c, d) Representative protein bands and quantification analyses of a Western blot for long MLCK, p-MLC-2/MLC-2, ZO-1, and occludin in Caco-2 cells transfected with pLKO.1-sh-HO-1 plasmid after pretreatment of ML-7 with or without TNF-*α* stimulation. All data presented were representative of three or more independent experiments, each with similar results. ^∗^*P* < 0.05 and ^∗∗^*P* < 0.01.

To further dissect the functional role of epithelial long MLCK in HO-1-mediated barrier loss restoration, Caco-2 cells were pretreated with ML-7, a long MLCK inhibitor. As shown in Figures 4(a) and 4(b), long MLCK protein expression and MLC-2 phosphorylation were markedly decreased, and epithelial ZO-1 and occludin expression was increased in HO-1 overexpression Caco-2 cell lines pretreated with ML-7 after TNF-*α* stimulation compared to the control group. However, relative to the control group, HO-1 shRNA completely reversed these effects after TNF-*α* stimulation, even in the presence of ML-7 (Figures 4(c) and 4(d)). These data indicated that the downstream MLCK/p-MLC-2 signaling pathway directly contributes to barrier loss, and HO-1 suppresses the activation of MLCK/p-MLC-2 signaling in a dependent manner.

### 3.5. Intestinal HO-1-Deficient Mice Contribute to Increased Intestinal Permeability after CCl_4_ Injection

**Figure 5:**
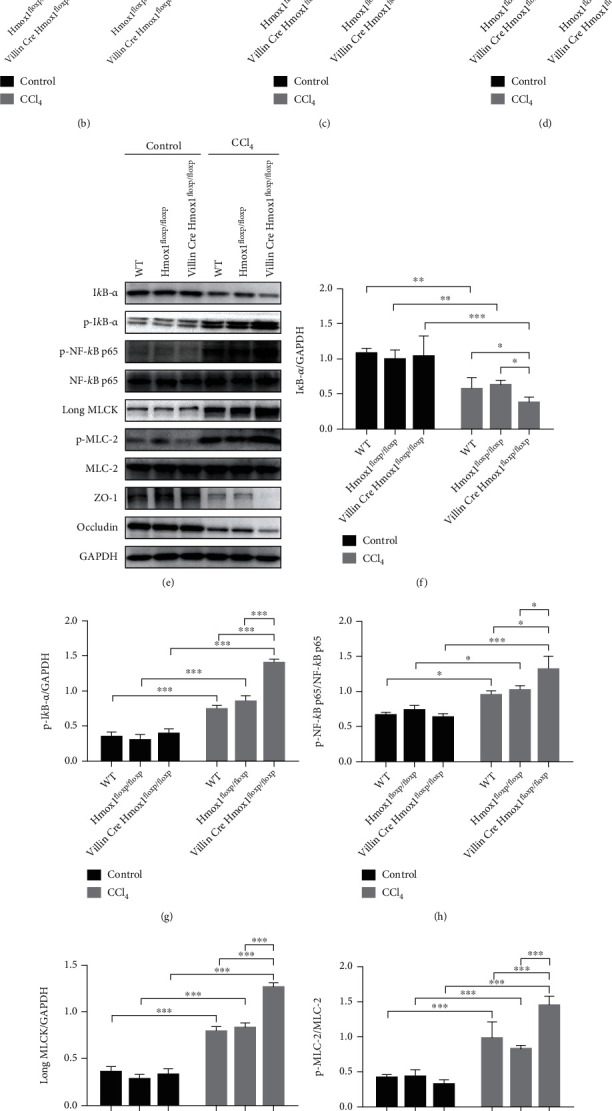
HO-1^−/−^ mice lose the protective effect against barrier loss after CCl_4_. (a) Representative colon sections after hematoxylin and eosin (H&E) staining (200x, scale bar = 10 *μ*m). (b) Colon pathology scores. (c) Colonic TNF-*α* levels (*n* = 3). (d) Colon length (cm) (*n* = 3). (e) Representative protein bands and quantification analyses of a Western blot for (f) I*κ*B-*α*, (g) p-I*κ*B-*α*, (h) p-NF-*κ*B p65/NF-*κ*B p65, (i) long MLCK, (j) p-MLC-2/MLC-2, (k) ZO-1, and (l) occludin in the colonic epithelia. All data presented were representative of three or more independent experiments, each with similar results. ^∗^*P* < 0.05, ^∗∗^*P* < 0.01, and ^∗∗∗^*P* < 0.001.

To further confirm the intestinal epithelial cell-specific function of HO-1 in mediating barrier loss, we used VillinCre Hmox1^floxp/floxp^ mice whose *hmox1* genes were conditionally knocked out in intestinal epithelial cells. WT and Hmox1^floxp/floxp^ mice were used as the controls for the experiments involving VillinCre Hmox1^floxp/floxp^ mice. HO-1^−/−^ mice showed more serious colonic mucosal injury, which was characterized by infiltration of inflammatory cells in colonic serosa and thickening of the colon wall (Figure 5(a)). Yet, no significant difference was observed in pathological colon scores between VillinCre Hmox1^floxp/floxp^ mice and WT or Hmox1^floxp/floxp^ mice after CCl_4_ challenge (Figure 5(b)). The TNF-*α* levels of the colon were increased (Figure 5(c), *P* < 0.001), and the length of the colon was decreased (Figure 5(d), *P* < 0.05) in VillinCre Hmox1^floxp/floxp^ mice, as compared to WT mice, after CCl_4_ administration. The expression of colonic epithelial TJ proteins (ZO-1 and occludin) was significantly reduced in VillinCre Hmox1^floxp/floxp^ mice, as compared to WT and Hmox1^floxp/floxp^ mice after CCl_4_ administration (Figures 5(e), 5(k), and 5(l); *P* < 0.05 and *P* < 0.01, respectively). Importantly, colonic epithelial I*κ*B-*α* degradation and phosphorylation, NF-*κ*B p65 activation and phosphorylation, long MLCK activation, and MLC-2 phosphorylation were significantly higher in HO-1^−/−^ mice than WT and Hmox1^floxp/floxp^ mice after CCl_4_ administration (Figures 5(e)–5(j)). These findings further demonstrated the crucial contributions of HO-1 in maintaining intestinal barrier integrity and the relative mechanisms in these processes.

### 3.6. HO-1/CO Repairing Intestinal Barrier Damage Is Protected against Liver Injury

**Figure 6:**
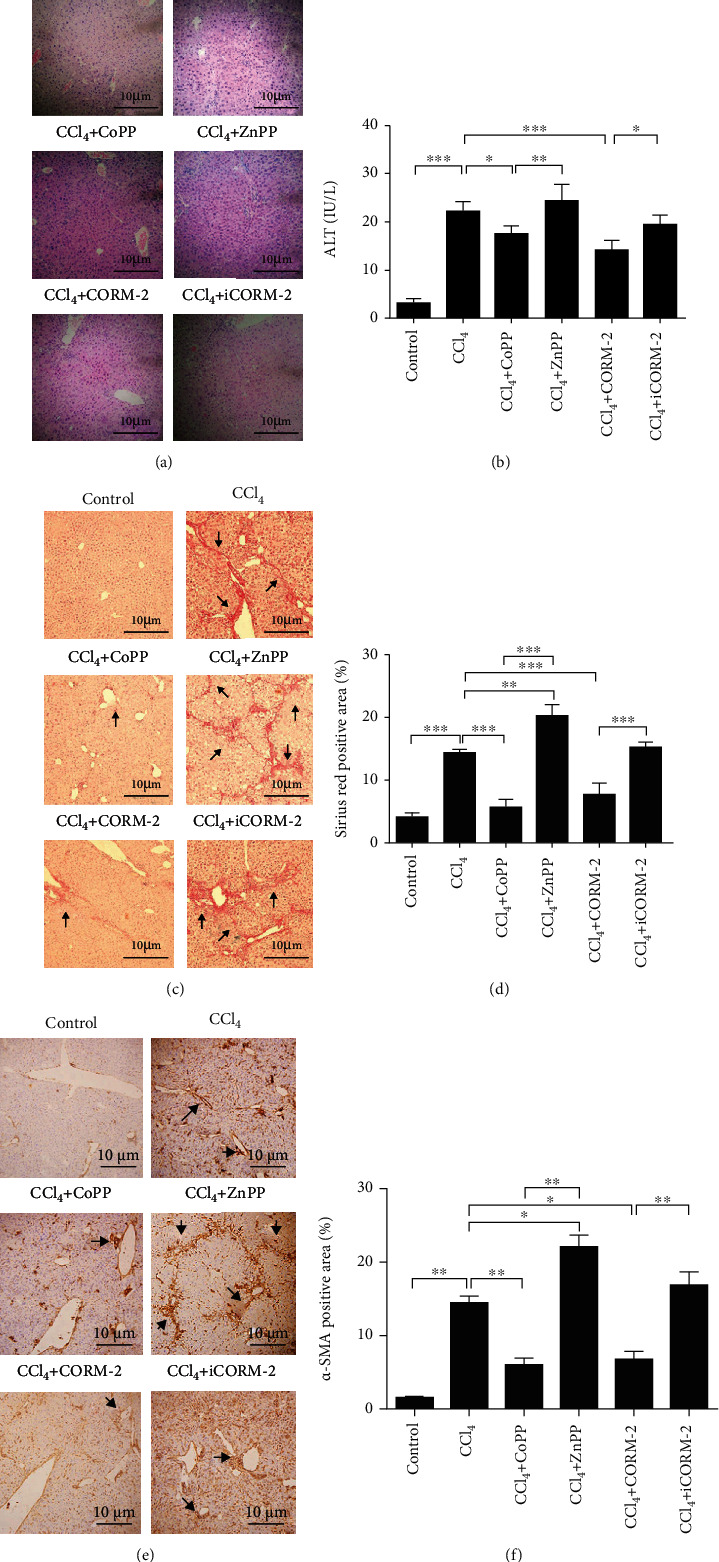
CoPP and CORM-2 repairing intestinal barrier damage diminished hepatic injury. Representative liver sections after hematoxylin and eosin (H&E) staining (a), Mayer-Sirius red staining (c), and immunohistochemistry staining for *α*-SMA (e) (200x, scale bar = 10 *μ*m), and serum ALT (*n* = 5) (b) levels; quantification of the Sirius red positive areas (d) and the *α*-SMA positive areas (f). Representative positive areas were indicated by black arrows. ^∗^*P* < 0.05, ^∗∗^*P* < 0.01, and ^∗∗∗^*P* < 0.001.

**Figure 7:**
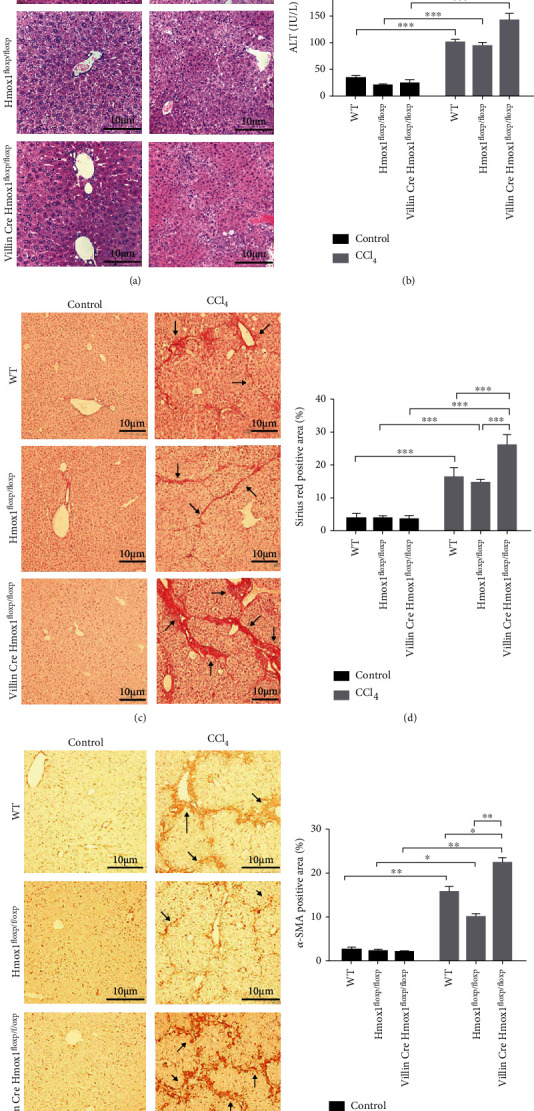
HO-1^−/−^ mice promote liver injury by aggravating intestinal mucosal barrier injury. Representative liver sections after hematoxylin and eosin (H&E) staining (a) (400x, scale bar = 10 *μ*m), Mayer-Sirius red staining (c), and immunohistochemistry staining for *α*-SMA (e) (200x, scale bar = 10 *μ*m) and serum ALT (*n* = 3) (b) levels; quantification of the Sirius red positive areas (d) and the *α*-SMA positive areas (f) are shown. Representative positive areas are indicated by black arrows. ^∗^*P* < 0.05, ^∗∗^*P* < 0.01, and ^∗∗∗^*P* < 0.001.

Finally, we verified the clinical significance of HO-1/CO repairing intestinal barrier damage in actual disease. Supplementary Table 1 shows liver fibrosis grades in each group. All control mice were Grade 0; Grade 3 fibrosis was only observed in the CCl_4_+ZnPP and CCl_4_+iCORM-2 groups. The distinct pathological changes in the liver, including the proliferation of fibrous tissue around the portal area (Figure 6(a)), the formation of a fibrous septum (Figure 6(a)), and the elevation of serum ALT levels (Figure 6(b), *P* < 0.001), collagen deposition (Figures 6(c) and 6(d), *P* < 0.001), and *α*-SMA expression (Figures 6(e) and 6(f), *P* < 0.001) were observed in the CCl_4_-treated group. The proliferation of hepatic fibrosis tissues (Figures 6(a), 6(c), and 6(d)), the expression of *α*-SMA (Figures 6(e) and 6(f)), and serum ALT levels (Figure 6(b)) in the CoPP and CORM-2 treatment groups were significantly lower than those of the CCl_4_ model group. The group treated with ZnPP showed disordered hepatic lobular structures and significantly increased fibrous tissue proliferation (Figure 6(a)), collagen deposits (Figures 6(c) and 6(d)), and *α*-SMA expression (Figures 6(e) and 6(f)) compared to the CCl_4_-treated group. There was no significant difference in hepatic fibrosis between the CCl_4_ and iCORM-2 groups (all *P* > 0.05) (Figure 6). In addition, VillinCre Hmox1^floxp/floxp^ mice showed disordered hepatic lobular structures, more fibrous tissue proliferation and collagen deposits, higher *α*-SMA expression in the liver, and higher serum ALT levels than WT and Hmox1^floxp/floxp^ mice after CCl_4_ exposure (Supplementary Table 2 and Figure 7). In summary, these data were consistent with the vital contributions of HO-1/CO to the intestinal barrier restoration pathway.

## 4. Discussion

Our data suggested that colonic mucosal injury, TNF-*α* production, TJ disruption, and epithelial NF-*κ*B p65/MLCK/p-MLC-2 signaling pathway activation are markedly decreased by exogenous upregulation of HO-1 or endogenous supplementation of CO after chronic CCl_4_ injection. The effects of TNF-*α* on TJ permeability and epithelial NF-*κ*B p65/MLCK/p-MLC-2 signaling pathway activation are attenuated in an HO-1-dependent fashion. Using intestinal HO-1-deficient mice further demonstrates the crucial contributions of HO-1 in maintaining intestinal barrier integrity and the relative mechanisms in these processes. Consistent with the above conclusions, alleviated hepatic fibrogenesis and serum ALT levels confirm the clinical significance of HO-1/CO repairing intestinal barrier injury. To the best of our knowledge, this is the first study on the functional linking of the intestinal HO-1/CO-NF-*κ*B/MLCK system to gut leakiness at different levels.

HO-1 and CO might be possible candidates to initiate intestinal barrier-restorative effects due to their anti-inflammation and antioxidative damage properties [27, 28]. However, they often used a kind of middle mechanism (e.g., the nuclear factor erythroid-2-related factor 2 (Nrf_2_)/HO-1/CO pathway) for regulating the intestinal barrier dysfunction [29, 30], and the direct effect of the HO-1-CO axis on intestinal barrier injury is poorly understood. Our data indicated that intestinal mucosal injury, TNF-*α* production, and TJ disruption are markedly attenuated by exogenous upregulation of HO-1 (CoPP) or endogenous supplement CO (CORM-2) after chronic CCl_4_ injection. As the major connection between intestinal epithelial cells, the TJ proteins in intestinal mucosa have an important role in maintaining the intestinal mucosa's mechanical barrier integrity and functions [1]. Decreased expression of TJ proteins leads to the increase of intestinal permeability, thus facilitating the entry of pathogens and toxic substances into the body [31–33]. TNF-*α* may have a central role in the complex chain reaction of cytokine-mediated intestinal mucosal injury [21, 22, 34]. Consistent with *in vivo* studies, our *in vitro* data showed that HO-1 dependently attenuates TJ disruption in the cell monolayers after TNF-*α* stimulation. Moreover, using intestinal HO-1^−/−^ mice, we further confirmed the vital role of intestinal-specific HO-1 in mediating barrier loss.

Previous studies reported that intestinal damage induces long MLCK expression by activating the NF-*κ*B signaling pathway [21, 35]. We demonstrated that intestinal I*κ*B-*α*, as an upstream inhibitor of NF-*κ*B, is degraded and phosphorylated, after which the NF-*κ*B p65 is activated and has a central role as a key transcription factor of barrier loss. We previously demonstrated that the inhibition of NF-*κ*B p65 contributes to stabilizing the intestinal barrier [13]. However, we provided no evidence that NF-*κ*B p65 directly mediates the intestinal TJ protein dysregulation. The presence of increased epithelial long MLCK expression and activity, which is mediated by NF-*κ*B p65, contributes to TJ dysregulation [14–16]. MLC-2 has a central role as a common final pathway of barrier disruption, and the phosphorylation of MLC-2 is the molecular basis for the increase of permeability of the intestinal barrier [1, 36]. This study shows that TNF-*α* strongly induces epithelial NF-*κ*B p65 phosphorylation, long MLCK expression, and MLC-2 phosphorylation and, consequently, increases the TJ disruption in the cell monolayers. Intestinal barrier disruption is facilitated by NF-*κ*B p65 expressed on intestinal epithelial cells. Moreover, NF-*κ*B p65-mediated activation of epithelial long MLCK and phosphorylation of MLC-2 in enterocytes and disruption of TJs contribute to reduced barrier function after TNF-*α* stimulation.

Many studies have suggested that the inhibition of the NF-*κ*B pathway and activation of the HO-1 pathway are anti-inflammatory mechanisms [37, 38], while only a few clearly clarified the crosstalk between them in regulating intestinal barrier loss. In the present study, CoPP and CORM-2 markedly alleviated colonic mucosal injury and TNF-*α* levels; upregulated TJ expression; and inhibited epithelial I*κ*B-*α* degradation and phosphorylation, NF-*κ*B p65 phosphorylation, long MLCK expression, and MLC-2 phosphorylation after CCl_4_. However, ZnPP and intestinal HO-1-deficient completely reversed these effects. Furthermore, HO-1 overexpression prevented TNF-*α*-induced TJ disruption, while HO-1 shRNA promoted TJ damage even in the presence of JSH-23 or ML-7. These data suggested that HO-1 dependently protects the intestinal barrier via inhibition of the NF-*κ*B p65/MLCK/p-MLC-2 pathway.

The above data confirmed the crucial contributions and mechanisms of HO-1/CO in maintaining intestinal barrier integrity. There is an emerging concept that disruption of intestinal barrier function has a central role in the initiation of acute liver injury and progression to chronic liver disease [6, 39]. Our study further verified the clinical significance of HO-1/CO repairing intestinal barrier injury by alleviating hepatic fibrogenesis and serum ALT levels. Intestinal epithelial barrier disruption can enhance or directly trigger IBD or dysbiosis [40, 41]; therefore, it is of great significance to study the effects of HO-1/CO maintaining intestinal barrier integrity on targeted treatment of IBD or intestinal microecological diseases.

In conclusion, this is the first study that suggested a link of the HO-1-CO axis in intestinal permeability by using a tissue or tissue-specific genetically modified mice and cell culture system. HO-1/CO regulates the intestinal barrier integrity through inhibition of the NF-*κ*B p65/MLCK/p-MLC-2 signaling pathway, and the HO-1/CO-NF-*κ*B/MLCK system in the intestine is a potential therapeutic target for barrier loss. Based on these findings, the significance of HO-1/CO maintaining intestinal barrier integrity should be tested in more clinical diseases with a leaky gut.

## Data Availability

Requests for data will be considered by the corresponding author 1 month after publication of this article.

## Abbreviations

LPS: Lipopolysaccharide
IBD: Inflammatory bowel disease
TJ: Tight junction
ZO-1: Zonula occludens 1
MLCK: Myosin light-chain kinase
MLC-2: Myosin light chain 2
NF-*κ*B: Nuclear factor-kappa B
TNF-*α*: Tumor necrosis factor *α*
HO-1: Heme oxygenase-1
CO: Carbon monoxide
CORM-2: Carbon monoxide-releasing molecule 2
CCl_4_: Carbon tetrachloride
WT: Wild type
CoPP: Cobalt protoporphyrin
ZnPP: Zinc protoporphyrin
iCORM-2: Inactivated-CORM-2
ALT: Alanine aminotransferase
I*κ*B-*α*: Inhibitor of nuclear factor-*κ*B *α*
Nrf_2_: Nuclear factor erythroid-2-related factor 2.

## Supplementary Materials

Supplementary Materials Supplementary Figure S1: validation of HO-1 expression in colonic epithelia after CCl_4_ modeling. (a) The representative protein bands and the quantification of a Western bolt for HO-1 in colonic epithelia. (b) Fold-change of HO-1 mRNA expression in the colon by qRT-PCR. All presented data were representative of three or more independent experiments, each with similar results. ^∗∗∗^*P* < 0.001. Supplementary Figure S2: the expression of HO-1 in Caco-2 cells transfected with the FUGW-HO-1 or pLKO.1-sh-HO-1 plasmid. Representative protein bands and quantification analyses of a Western blot for HO-1 in Caco-2 cells transfected with the FUGW-HO-1 plasmid (a) or pLKO.1-sh-HO-1 plasmid (b) with or without TNF-*α* stimulation. All presented data were representative of three or more independent experiments, each with similar results. ^∗∗∗^*P* < 0.001. Supplementary Figure S3: schematic of the construction of the HO-1^−/−^ mice with conditional knockout of HO-1 in intestinal epithelial cells. (a) The principle of intestinal *hmox1* conditional knockout mice. The CRISPR/Cas9 technology was used to cut the target gene's DNA intron and provide the homologous template donor. FloxP was inserted at both ends of the specific exon through homologous recombination and DNA repair. Hmox1-FloxP mice were crossed with Cre mice, and the specific exon of *hmox1* was deleted. Thus, *hmox1* does not translate and produces a frame-shift mutation, and the HO-1 protein is inactivated, thereby achieving conditional knockout of the *hmox1* gene. (b, c) The transcription ability of small guide RNA (sgRNA) was evaluated according to the cutting activity of the Cas9/gRNA complex. Finally, the sgL5 and sgR8 sites were chosen. (d) Schematic of the Cas9/gRNA complex enzyme cutting the DNA intron of the *hmox1* gene. (e) The FloxP site insert results were evaluated by PCR. (f) The FloxP site insert results were evaluated by sequencing. Table S1. Pathological grading of liver fibrosis in each proups. Table S2. Pathological grading of liver fibrosis in each groups
