# Supplementary material for: HO-1/CO Maintains Intestinal Barrier Integrity through NF-κB/MLCK Pathway in Intestinal HO-1−/− Mice
Source: Oxid Med Cell Longev. 2021 May 19;2021:6620873. doi: 10.1155/2021/6620873 (PMC8159651; doi:10.1155/2021/6620873)

a

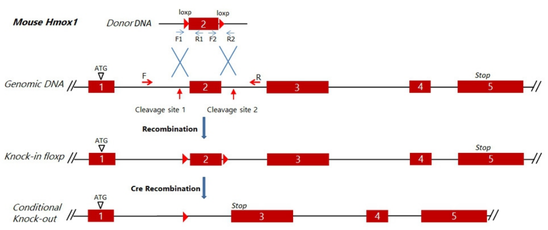

b

sgRNA	sites	Target sequences(5'-3')
sgL1	5'	CTGTTTGCATGGTTAAGACGG
sgL2	5'	GACTGACATCTGTTTGCATGG
sgL3	5'	GATTCTGCCTGTGGTTTTGGG
sgL4	5'	GAATAGGGATTCTGCCTGTGG
sgR1	3'	AAGGTGACTTCCTTGGACAGG
sgR2	3'	GTAATAAGGTGACTTCCTTGG
sgR3	3'	CACAGTATCAATGTAATAAGG
sgR4	3'	TTAACCATCCTGCTGCACTGG

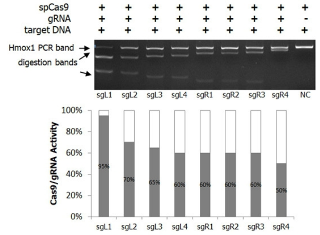

c

sgRNA	sites	Target sequences(5'-3')
sgL5	5'	TCAGTCATTAAGTACTATGGG
sgL6	5'	TATTGTCCCCAAAACCACAGG
sgL7	5'	TAAAGGGGAGGCAAGGTATGG
sgL8	5'	CCAACCACTATGCTCAAAGGG
sgR5	3'	CTTATTACATTGATACTGTGG
sgR6	3'	GAGAAGATGTGATGCGGGTGG
sgR7	3'	AGTGCAGCAGGATGGTTAAGG
sgR8	3'	GGAACATGTATTCTGAGTTGG

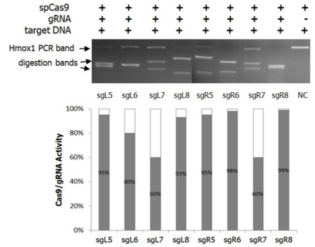

d

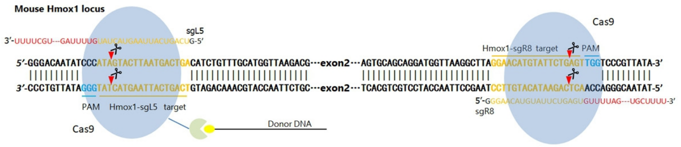

e

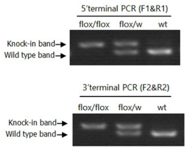

f

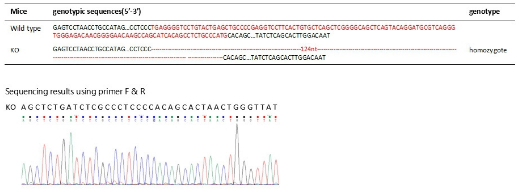

Supplement: Supplementary Materials — Supplementary Figure S1: validation of HO-1 expression in colonic epithelia after CCl4 modeling. (a) The representative protein bands and the quantification of a Western bolt for HO-1 in colonic epithelia. (b) Fold-change of HO-1 mRNA expression in the colon by qRT-PCR. All presented data were representative of three or more independent experiments, each with similar results. ∗∗∗P < 0.001. Supplementary Figure S2: the expression of HO-1 in Caco-2 cells transfected with the FUGW-HO-1 or pLKO.1-sh-HO-1 plasmid. Representative protein bands and quantification analyses of a Western blot for HO-1 in Caco-2 cells transfected with the FUGW-HO-1 plasmid (a) or pLKO.1-sh-HO-1 plasmid (b) with or without TNF-α stimulation. All presented data were representative of three or more independent experiments, each with similar results. ∗∗∗P < 0.001. Supplementary Figure S3: schematic of the construction of the HO-1−/− mice with conditional knockout of HO-1 in intestinal epithelial cells. (a) The principle of intestinal hmox1 conditional knockout mice. The CRISPR/Cas9 technology was used to cut the target gene's DNA intron and provide the homologous template donor. FloxP was inserted at both ends of the specific exon through homologous recombination and DNA repair. Hmox1-FloxP mice were crossed with Cre mice, and the specific exon of hmox1 was deleted. Thus, hmox1 does not translate and produces a frame-shift mutation, and the HO-1 protein is inactivated, thereby achieving conditional knockout of the hmox1 gene. (b, c) The transcription ability of small guide RNA (sgRNA) was evaluated according to the cutting activity of the Cas9/gRNA complex. Finally, the sgL5 and sgR8 sites were chosen. (d) Schematic of the Cas9/gRNA complex enzyme cutting the DNA intron of the hmox1 gene. (e) The FloxP site insert results were evaluated by PCR. (f) The FloxP site insert results were evaluated by sequencing. Table S1. Pathological grading of liver fibrosis in each proups. Table S2. Pathological gra [file 6620873.f1.zip › Supplementary Fig S3.pdf]
